# Supplementary material for: Zebrafish Avatars: Toward Functional Precision Medicine in Low-Grade Serous Ovarian Cancer
Source: Cancers (Basel). 2024 May 9;16(10):1812. doi: 10.3390/cancers16101812 (PMC11120355; doi:10.3390/cancers16101812)
Supplement: Supplementary file 1 [file cancers-16-01812-s001.zip › cancers-2949875-supplementary.pdf]

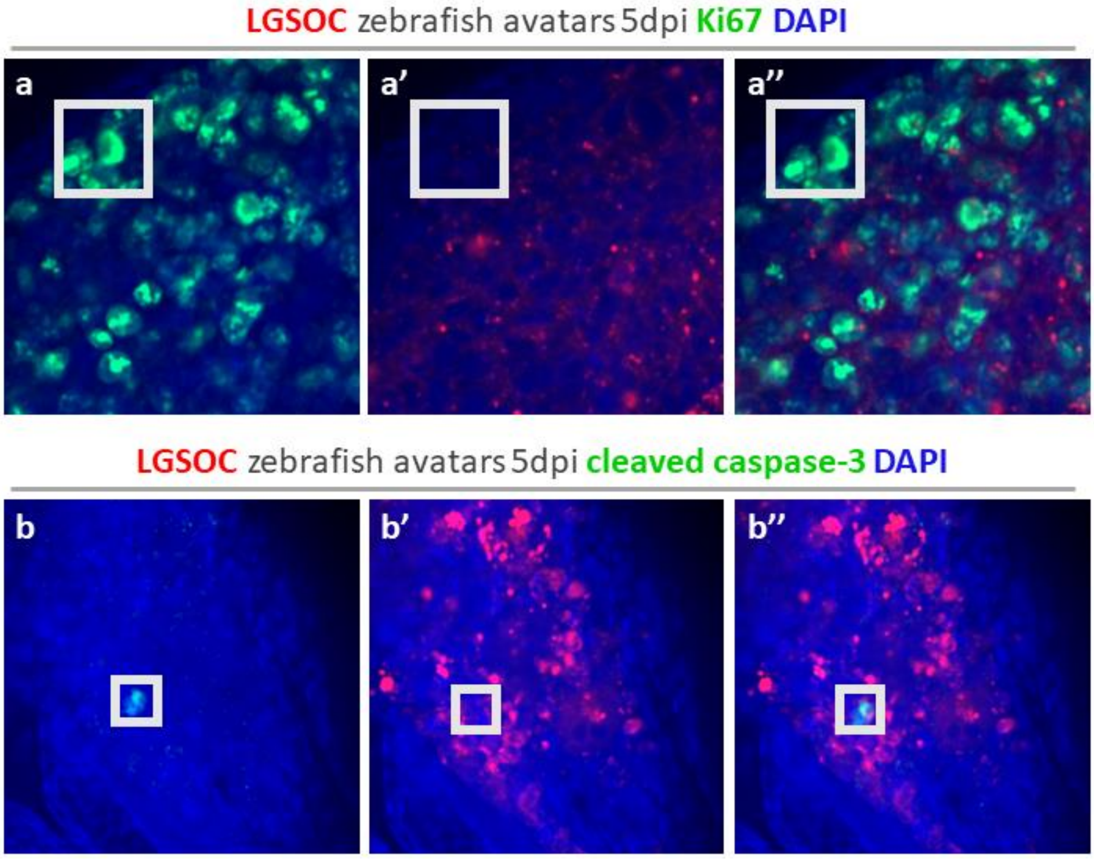

Figure S1: Analysis of merged channels for vybrant DiI staining and immunofluorescent markers Ki67 (a-a’’) and cleaved caspase-3 (b-b’’). *Cells with expression of Ki67 (a, green) are expected to overlap with vybrant DiI staining (a’, red) in composite (a’’). However, the white box indicate cells with Ki67 expression without vybrant DiI staining. Cells with expression of cleaved caspase-3 (b, green) should not overlap with vybrant DiI (b’, red) in composite (b’’). The white box indicated a cell with caspase-3 with clear overlap with red fluorescent staining.*

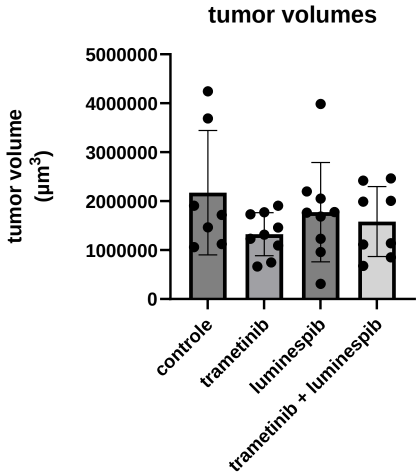

Figure S2: Tumor volume end point measurements after 4 days of treatment*. Tumor volume (µm3) of different treatment conditions are presented as AVG ± SEM and each dot represents one xenograft. Multiple comparisons between treatment groups w were determined by One-way ANOVA, Tukey procedure was used to adjust p-values. No significant differences were observed between treatment conditions.*
